# Editorial: A year in review: discussions in cancer endocrinology

**DOI:** 10.3389/fendo.2023.1289156

**Published:** 2023-09-20

**Authors:** Madson Almeida, Javad Sharifi-Rad, Veronica Vella

**Affiliations:** ^1^ Divisione of Endocrinology and Metabolism, University of Sao Paulo Medical School, Sao Paulo, Brazil; ^2^ Facultad de Medicina, Universidad del Azuay, Cuenca, Ecuador; ^3^ Endocrinology, Department of Clinical and Experimental Medicine, University of Catania, Garibaldi-Nesima Hospital, Catania, Italy

**Keywords:** cancer endocrinology, prognosis, neuroendocrine tumours (NETs), nonalcoholic fatty liver disease (NAFLD), hyperthyroidism, insulin resistance, somatostatin analogue (SSA)

The Cancer Endocrinology field is continuously evolving; therefore, we tried to understand developments and perspectives on articles that have attracted attention throughout the year. Relevant papers add a significant contribution to the topic and examine the association between metabolic parameters and the risk of cancer (Wang et al.) as well as several prognostic factors associated with cancer such as the number of metastatic lymph nodes in patients with papillary thyroid cancer (Sun et al.), the presence of hyperthyroidism (Xu et al.), or a glycolytic and immune signature (Guo et al.).

This Research Topic includes eleven original articles focusing mainly on prognostic aspects of several cancer ranging from solid (thyroid, colon, prostate and neuroendocrine) tumours to leukemia. Different issues relating to prognosis have been taken into consideration. A meta-analysis was conducted in order to evaluate the effects of triglyceride glucose (TyG) index as a predictor of insulin resistance and the risk of cancer (Wang et al.). Six observational studies with 992292 participants were used to demonstrate that a high TyG index increased the risk of cancer in both Asia and Europe. Indeed, the association of environmental factors, including obesity (mainly visceral obesity) metabolic syndrome (MetS), and nonalcoholic fatty liver disease (NAFLD), the hepatic manifestation of MetS, has been implicated as risk factors for gastroenteropancreatic neuroendocrine neoplasms (GEP-NENs) (Barrea et al.). The authors reported that the worsening of clinicopathological characteristics in GEP-NET was associated with a higher presence of MetS, NAFLD, evaluated by fatty liver index (FLI), and visceral adiposity dysfunction assessed by visceral adiposity index (VAI).

Another parameter that has been considered in the prognosis of anaplastic thyroid cancer (ATC) patients is age (Kong et al.). Anaplastic cancer represents a small subset of thyroid tumours consisting of undifferentiated cells with a median survival rate of 5 months and a 1-year-survival rate of <20%. The authors confirmed that age at diagnosis influences the prognosis of ATC patients and calculated that the high-point age at diagnosis was 70 years. Thus, they associated the age > 70 years with a poor prognosis.

Observational studies have shown that hyperthyroidism may increase the risk of cancer, but its causal effect has not been investigated. In their paper, Xu et al. conducted a two-sample Mendelian randomisation (MR) study to explore the associations between genetic predisposition to hyperthyroidism and nine common types of cancer, including prostate, lung, breast, colon, leukemia, brain, skin, bladder, and esophagus cancer. A growing body of evidence suggests that thyroid hormones play important roles in regulating cellular processes, including those relevant to cancer development and progression. Results of Xu et al. paper provided evidence of a causal relationship between hyperthyroidism and the risk of prostate cancer, rectal cancer, and leukaemia and suggested a negative causal relationship between hyperthyroidism and prostate cancer.

It is widely acknowledged that aberrant glycolytic metabolism in cancer is related to tumour progression and acidifies the tumour microenvironment (TME). Through the Glycolysis Score containing numerous genes and TME Score including three immune cells (macrophage M2, plasma cells and regulatory T cells -Tregs), Guo et al. constructed the Glycolysis-TME Classifier demonstrating that it was more accurate in predicting the prognosis of patients than the current biomarkers particularly in the management of patients with prostate cancer. As regards to the staging of a rare tumour, Cuthbertson et al. suggested that ^68^Ga-DOTA PET/CT imaging is more accurate and guides the treatment of patients with both sporadic and familial pancreatic neuroendocrine tumours (panNETs) and newly diagnosed or recurrent disease, mainly where surgical resection or treatment with peptide receptor radionuclide therapy (PRRT) is considered. On the same topic regarding the NETs, Lenotti et al. explored the outcome measures (progression-free survival, PFS; overall survival, OS; overall response rate and safety) and prognostic factors associated with PFS in patients with metastatic lung NET treated with first-line somatostatin analogues (SSA)-monotherapy (octreotide or lanreotide). Through a retrospective study, they observed a longer PFS and OS suggesting that SSAs could be effective as a first-line approach in the management of patients with progressive, metastatic pulmonary NET.

Another rare and aggressive tumour with a very poor prognosis is adrenocortical carcinoma (ACC). The current standard treatment includes complete surgical resection for localised respectable disease and systemic therapy with mitotane. However, the efficacy of systemic therapy in ACC is very limited, with high rates of toxicities. Two original papers were focused on this issue. Notably, Rossini et al. explored the possible *in vitro* effects of tamoxifen on ACC cell viability and investigated the additive/synergic cytotoxic activity of tamoxifen and progesterone in ACC experimental cell models. In their paper, Shen et al. identified and validated anm6A based signature, which can be used as an independent prognostic factor in evaluating the prognosis of ACC patients.

Finally, the study of Kucka et al. examined the expression of the PIK genes that control the production of phosphoinositides and the possible role of these signalling molecules on PRL-secreting lactotrophs of the anterior pituitary gland. Their results revealed that inhibition of PI4Ks, but not other kinases, is able to block PRL release downstream of calcium signalling.

Taken together these findings from original papers underline the necessity to further deepen aspects related to the pathogenetic mechanisms of many endocrine tumours in order to find new ways to better stratify patients from a prognostic point of view. Hopefully ongoing and future studies will be able to achieve significant targets.

## Author contributions

JS-R: Writing – review & editing. MA: Writing – review & editing. VV: Writing – review & editing.

